# Association between allergic conditions and risk of prostate cancer: A Prisma-Compliant Systematic Review and Meta-Analysis

**DOI:** 10.1038/srep35682

**Published:** 2016-10-21

**Authors:** Jianguo Zhu, Jukun Song, Zezhen Liu, Jin Han, Heng Luo, Yunlin Liu, Zhenyu Jia, Yuanbo Dong, Wei Zhang, Funeng Jiang, Chinlee Wu, Zaolin Sun, Weide Zhong

**Affiliations:** 1 Department of Urology, Guangdong Key Laboratory of Clinical Molecular Medicine and Diagnostics, Guangzhou First People’s Hospital, Guangzhou Medical University, Guangzhou 510180, China; 2 Urology Key Laboratory of Guangdong Province, The First Affiliated Hospital of Guangzhou Medical University, Guangzhou Medical University, Guangzhou, 510230, China; 3 Department of Urology, Guizhou Provincial People’s Hospital, Guizhou, 550002, China; 4 Department of oral and maxillofacial surgery, Guizhou Provincial People’s Hospital, Guizhou, 550002, China; 5 Department of Respiratory & Critical Care medicine, Guizhou Provincial People’s Hospital, Guizhou, 550002, China; 6 College of Agricultural and Environmental Sciences, University of California, Davis, CA 95616, USA; 7 Departments of Pathology and Urology, Massachusetts General Hospital and Harvard Medical School, Boston, MA 02114, USA

## Abstract

Association between allergic conditions and prostate cancer risk has been investigated for many years. However, the results from available evidence for the association are inconsistent. We conducted a meta-analysis to evaluate the relationship between allergic conditions (asthma, atopy, hay fever and “any allergy”) and risk of prostate cancer. The PubMed and Embase databases were searched to screen observational studies meeting our meta-analysis criteria. Study selection and data extraction from included studies were independently performed by two authors. Twenty studies were considered eligible involving 5 case-control studies and 15 cohort studies. The summary relative risk (RR) for developing prostate cancer risk was 1.04 (95%CI: 0.92–1.17) for asthma, and 1.25 (95%CI: 0.74–2.10) for atopy, 1.04 (95%CI: 0.99–1.09) for hay fever, 0.96 (95%CI: 0.86–1.06) for any allergy. In the Subgroup and sensitivity analysis, similar results were produced. Little evidence of publication bias was observed. The present meta-analysis of observational studies indicates that no indication of an association between allergic conditions and risk of prostate cancer was found, and the meta-analysis does not support neither the original hypothesis of an overall cancer protective effect of allergic conditions, nor that of an opposite effect in the development of prostate cancer.

Prostate cancer is one of most frequently diagnosed cancer in Western countries and is the second most common cancer in men, following lung cancer, worldwide^1^,^2^. In 2015, up to 220,800 men were diagnosed with prostate cancer, and 27,540 men will die of it in the United States^1^. The incidence and mortality rates of prostate cancer vary markedly among different ethnic groups, with the lowest rates found in China and other parts of Asia and the highest rates detected in Western populations^3^. The etiology of prostate cancer comprises multiple factors. Established risk factors for prostate cancer included obesity, old age, ethnicity, androgen, and environmental factor, androgen, and diet^4^,^5^,^6^,^7^. There are some studies addressing systematic inflammation conditions and immune response that contribute to prostate tumorigenesis^8^,^9^.

Allergy-related carcinogenesis is a topic of interest but has generated considerable controversy. Considering the impact of the prostate cancer risk potentially resulting from allergic diaseases, a number of studies have explored the association between allergic diseases and prostate cancer risk^10^,^11^,^12^,^13^,^14^,^15^,^16^,^17^,^18^,^19^. However, individual studies have yielded inconsistent or conflicting findings, possibly caused by limitation associated with an individual study. In a previous meta-analysis of studies (only included nine studies), asthma, hay fever and allergy were not associated with prostate cancer risk, but not for atopy^20^. Two additional studies were not included in the meta-analysis^21^,^22^. Subsequent publishing studies have also found inconsistent results of associations between allergic disease and prostate cancer risk, with positive association^23^,^24^,^25^,^26^, inverse association^27^, and no association^28^,^29^,^30^,^31^. To shed light on these contradictory results and to more precisely evaluate the relationship among allergic diseases and prostate cancer, we performed an up-dated meta-analysis of published studies. Nevertheless, clarifying a relationship might emphasize the importance of considering additional preventative methods for prostate cancer. The study was reported in accordance with the Preferred Reporting Items for Systematic Reviews^32^.

## Results

### Literature search

**Figure 1:**
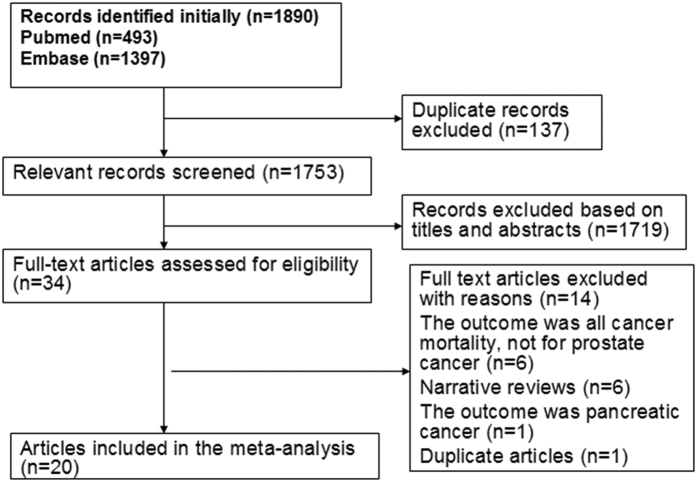
Flow diagram of selection of studies included in the meta-analysis.

Following the development of our search strategy, 1,890 records were initially retrieved. After excluding the duplicates and articles that did not meet the inclusion criteria, we reviewed 34 possible relevant studies in full-text. A total of 14 studies were excluded for the following reasons: Two articles were covered the same population^27^,^31^; six articles were narrative reviews^33^,^34^,^35^,^36^,^37^,^38^; one article reported the association allergic disorders and pancreatic cancer^39^; and six articles were not related to outcome of interest^40^,^41^,^42^,^43^,^44^,^45^. Finally, 20 studies that met inclusive criteria were included in the meta-analysis (Fig. 1).

### Study characteristics

The Tables 1 and 2 shows the descriptive data for all included studies. A total of 20 studies, comprising 5 case-control studies with 2,924 incident cases and 7,175 controls and 15 cohort studies including 16,526 cases and 1,681,562 pariticipants, contributed to the meta-analysis. These studies were published from 1985 to 2015. The number of prostate cancer patients ranged from 1 to 6,294 in the case-control studies and from 10 to 1,936 in the cohort studies. Ten studies were conducted in Europe^10^,^12^,^13^,^14^,^17^,^23^,^24^,^28^,^29^,^30^, six in the North America^11^,^16^,^18^,^19^,^22^,^31^, two in Australia^15^,^25^ and two in Asia^21^,^26^. Eighteen studies reported findings for prostate cancer incidence^10^,^11^,^12^,^13^,^14^,^16^,^17^,^18^,^19^,^21^,^22^,^23^,^24^,^25^,^26^,^28^,^29^,^31^, whereas only two studies reported results for prostate cancer mortality^16^,^30^. We included a total of 5,757 prostate cancer deaths, 16,526 prostate cancer cases in the meta-analysis. The maximum numbers (1,102,247) of participants were from the Canada prospective study and minimum number (1,522) were from Australia prospective study in the cohort studies, while the maximum incident patients (1,936) were from the Canada Montreal PROtEuS study and minimum incident patients (10) were from Japan study in the case-control studies. The exposure categories that were measured were: 1) asthma; 2) hay fever (rhinitis); 3) atopy; and 4) any allergy (atopy and/or asthma, hay fever, or other allergic disease). For the assessment of allergic conditions, allergen-specific IgE measurement, skin prick testing, self-reported questionnaire, interviews by medical staff, and hospital discharge register were employed. For the assessment of prostate cancer, fourteen studies reported that cancers were histologically confirmed or were identified from national/regional cancer registeries, which we assumed verified the cancer pathologically. The remaining studies based their cancer diagnoses on different criteria; admission/discharge diagnoses; an automated general practice database; a linked national death register; review of hospital and nursing home recorder and death certificates. Six studies were designed to evaluate OR^10^,^17^,^18^,^19^,^22^,^31^, five evaluated HR^15^,^25^,^26^,^29^,^30^, three evaluated RR^11^,^16^,^21^,^28^, while five studies compared observed cancer incidence rates in cohort of patients with asthma or hay fever/allergic rhinitis against expected numbers estimated from population-based cancer registries and their effect estimates were standardized incidence ratio (SIR)^13^,^14^,^23^,^24^ or standardized mortality ratio(SMR)^12^.

Adjusted effect estimates could be determined for most cohort and case-control studies. Most risk estimates were adjusted for age (n = 12)^10^,^11^,^15^,^17^,^18^,^19^,^22^,^25^,^26^,^28^,^30^,^31^, smoking (n = 10)^10^,^11^,^15^,^16^,^17^,^18^,^19^,^25^,^28^,^29^ and body mass index (n = 8)^10^,^15^,^16^,^17^,^25^,^28^,^29^,^30^. Some studies were also controlled for alcohol consumption (n = 5)^10^,^16^,^17^,^25^,^29^ and race (n = 3)^16^,^19^,^28^, but few studies were adjusted for family history of prostate cancer (n = 2), total energy intake (n = 1)^25^, and intake of vegetable and red meat (n = 1)^16^. None of the studies were adjusted for exposure to heavy metals and androgen.

The methodological quality of the included studies was generally good. The NOS scores ranged from five to seven (Table 3). The median NOS score was 6.0.

### Asthma and risk of prostate cancer

**Figure 2:**
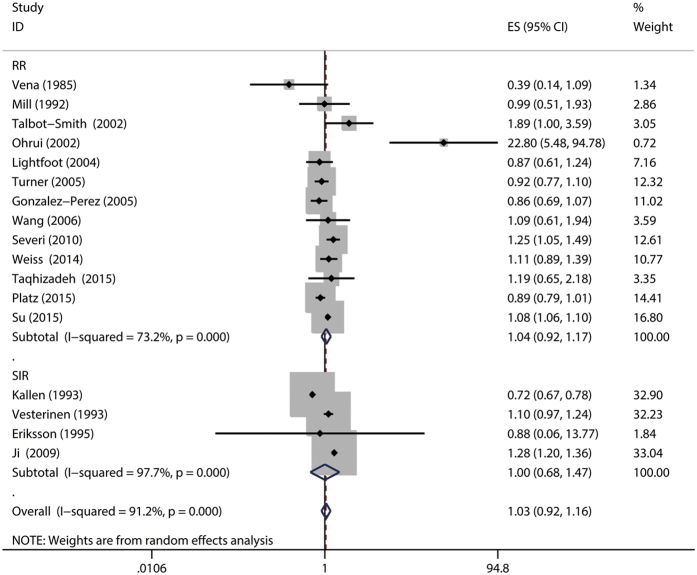
Forest plot of asthma and relative risk/standardized incidence ratio of prostate cancer.

**Figure 3:**
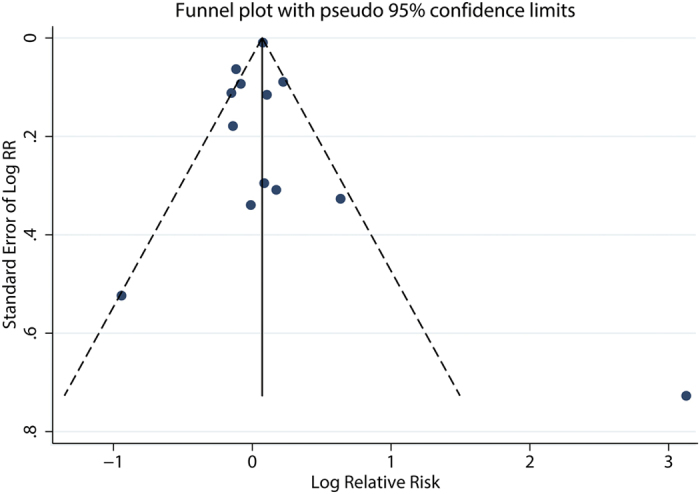
Funnel plot of asthma and relative risk of prostate cancer.

The association of asthma with prostate cancer was investigated in 17 studies^10^,^11^,^12^,^13^,^14^,^15^,^16^,^17^,^18^,^21^,^22^,^24^,^25^,^26^,^28^,^30^,^31^. Thirteen studies reported HR/RR/OR^10^,^11^,^15^,^16^,^17^,^18^,^21^,^22^,^25^,^26^,^28^,^30^,^31^, while four studies reported the SIR/SMR^12^,^13^,^14^,^24^. The combined relative risk was 1.04 (95%CI: 0.92–1.17), with significant heterogeneity (P_for heterogeneity_ = 0.000; *I*^2^ = 73.2%), while the pooled SIR was 1.00 (95%CI: 0.68–1.47), with significant heterogeneity (P_for heterogeneity_ = 0.000; *I*^2^ = 97.7%) (Fig. 2). In subgroup and sensitivity analysis, the results showed basically consistent with the overall analysis (Table 4). When we stratified the analysis by geographic region, the pooled RR was 0.95 (95%CI: 0.81–1.12) for studies conducted in North America, 0.90 (0.81–1.00) for studies conducted in Europe, and 4.55 (0.23–89.94) for studies conducted in Asia. In the subgroup analysis stratified by NOS quality, the combined RR was 1.90 (95%CI: 0.29–12.61) for low quality studies and 1.02 (95%CI: 0.93–1.13) for high quality studies. We restricted each analysis to study design, the combined RR was 1.02 (95%CI: 0.91–1.15) among case-control studies and 1.20 (94%CI: 0.69–2.09) among cohort studies, respectively. Nevertheless, when we stratified the analysis by adjusted for age, race, BMI, cigarette smoking and alcohol drinking, asthma was also not associated with risk of prostate cancer. In a sensitivity analysis, similar results were observed, which ranged from 1.01 (95%CI: 0.88–1.16) with significant heterogeneity (P_for heterogeneity_ = 0.000, *I*^2^ = 73.7%) (excluding the study by Severi G *et al.*^25^) to 1.07 (95%CI: 0.93–1.23) with significant heterogeneity (P_for heterogeneity_ = 0.000, *I*^2^ = 69.2%) (excluding the study by Platz *et al.*^28^). Only four of studies on asthma specially investigated advanced prostate cancer risk (advanced prostate cancer was defined as T3-4 and PSA > 50 ng/ml or Gleason grade ≥ 8) and asthma^25^,^28^, the summary RR was 0.86 (95%CI: 0.54–1.37), with significant heterogeneity (P_for heterogeneity_ = 0.079; *I*^2^ = 67.6%). Egger funnel plot asymmetry test (P = 0.865) and Begg rank correlation test (P = 0.502) were performed to assess publication bias and the funnel plot symmetry (Fig. 3) was examined. Finally, no proof of publication bias was obtained. Because of limited number of studies, we fail to conduct subgroup and sensitivity, publication analysis for studies reported the SIR or SMR.

### Hay fever and risk of prostate cancer

**Figure 4:**
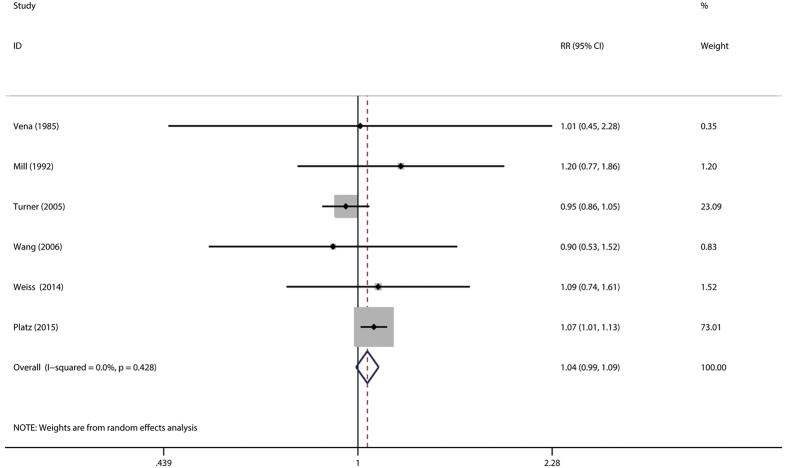
Forest plot of hay fever and relative risk of prostate cancer.

The association of hay fever with prostate cancer was examined in 7 studies^10^,^11^,^16^,^18^,^23^,^28^,^31^. The six studies reported RR/OR^10^,^11^,^16^,^18^,^28^,^31^ (Fig. 4), while SIR was reported in only one study, which reported a positive association between hay fever/allergic rhinitis and prostate cancer risk^23^. The combined relative risk was 1.04 (95%CI: 0.99–1.09), with low heterogeneity (P_for heterogeneity_ = 0.428; *I*^2^ = 0.0%). Subgroup and sensitivity analysis produced similar results (Table 4). In subgroup analysis stratified by geographic region, asthma was not associated with risk of prostate cancer (RR: 0.97; 95% CI: 0.88–1.06) in the four studies in North Ameria, but not in the two studies in Europe (RR:1.07; 95%CI: 1.01–1.13). Compared with a low NOS score (RR = 1.02, 95%CI: 0.94–1.11), the association was higher among studies with high NOS score (RR = 1.15, 95% CI: 0.78–1.70). When we restricted each analysis to study design, the combined RR was 1.03 (95%CI: 0.93–1.14) among case-control studies and 1.02 (95%CI: 0.76–1.36) cohort studies, respectively. When the analysis was restricted to studies adjusted for race, BMI and cigarette smoking, we found no association between asthma and prostate cancer, but not for studies adjusted for age and alcohol drinking. In a sensitivity analysis, similar results were observed, which ranged from 0.97 (95%CI: 0.88–1.06) with low heterogeneity (P_for heterogeneit_ = 0.360, *I*^2^ = 9.0%) (excluding the study by Platz *et al.*^28^) to 1.07 (95%CI: 1.01–1.13) with significant heterogeneity (P_for heterogeneit_ = 0.025, *I*^2^ = 58.4%) (excluding the study by Turne *et al.* which reported the association between asthma and mortality of prostate cancer^16^). Both the Begg rank correlation test (P = 0.707) and the Egger linear regression test (P = 0.775) in the meta-analysis indicated no significant publication bias. Because number of included studies less than 10, we did not perform the funnel plot.

### Atopy and risk of prostate cancer

**Figure 5:**
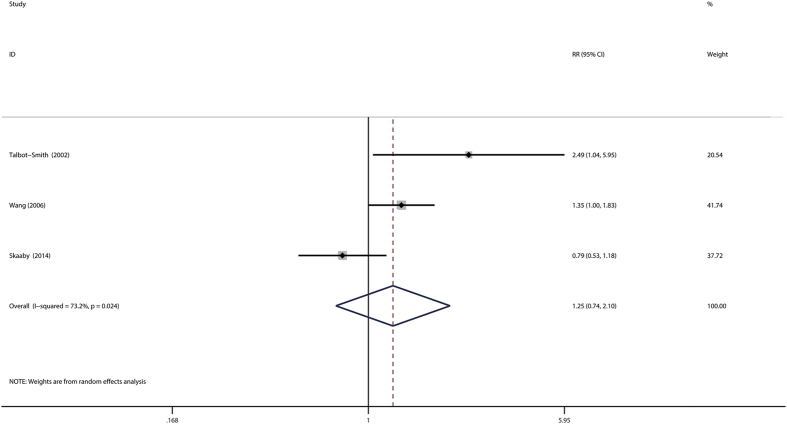
Forest plot of atopy and relative risk of prostate cancer.

The association of atopy with prostate cancer was investigated in 3 studies^10^,^15^,^29^. The combined relative risk was 1.25 (95%CI: 0.74–2.10), with notable heterogeneity (P_for heterogeneity_ = 0.024; *I*^2^ = 73.2%) (Fig. 5). In subgroup and sensitivity analysis, the results showed basically consistent with the overall analysis (Table 4). The include studies achieved six or more stars and considered to be of high quality, so the result was consistent with overall analysis. When the analysis was restricted to studies adjusted for age, race, BMI, alcohol drinking and cigarette smoking, we found no association between asthma and prostate cancer. In a sensitivity analysis, similar results were observed, which ranged from 1.05 (95%CI: 0.62–1.77) with low heterogeneity (P_for heterogeneity_ = 0.036, *I*^2^ = 77.6%) (excluding the study by Talbot-Smith *et al.*^15^) to 1.59 (95%CI: 0.93–2.71) with significant heterogeneity (P_for heterogeneity_  = 0.193, *I*^2^ = 41.0%) (excluding the study by Skaaby *et al.*^29^). Both Egger’s test (P = 1.000) and Bgger’s test (P = 0.761) showed no publication bias. Because number of included studies less than 10, we did not perform the funnel plot.

### Any allergy and risk of prostate cancer

**Figure 6:**
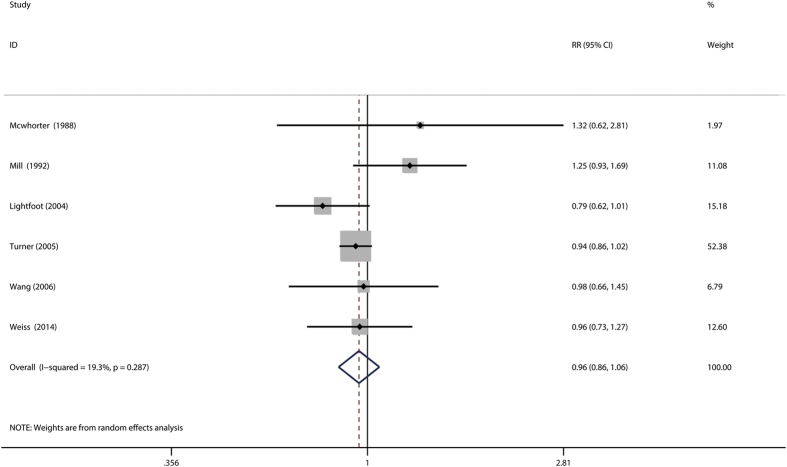
Forest plot of any allergy and relative risk of prostate cancer.

The association of asthma with prostate cancer was investigated in 6 studies^10^,^11^,^16^,^19^,^22^,^31^. The combined relative risk was 0.96 (0.86–1.06), with low heterogeneity (P_for heterogeneity_ = 0.287; *I*^2^ = 19.3%). Subgroup and sensitivity analysis yielded similar result (Fig. 6). In subgroup and sensitivity analysis, the results showed basically consistent with the overall analysis (Table 4). When we stratified the analysis by geographic region, the pooled RR was 0.96 (95%CI: 0.84–1.10) for 5 studies conducted in North America, 0.98 (95%CI: 0.66–1.45) for 1 studies conducted in Europe. Stratifying by study design, the combined RR was 1.06 (95%CI: 0.84–1.33) among case-control studies and 0.88 (95%CI: 0.75–1.04) among cohort studies, respectively. Compared with a low NOS score (SMR = 2.08, 95%CI: 0.73–5.91), the association was significant among studies with high NOS score (OR = 1.51, 95%CI: 1.14–1.98). In subgroup analysis adjusted for risk factors, including age, race, smoking and alcohol drinking, the results was consistent with overall analysis. In a sensitivity analysis, similar results were observed, which ranged from 0.93 (95%CI: 0.86–1.00) with low heterogeneity (P_for heterogeneity_ = 0.612, *I*^2^ = 0.0%) (excluding the study by Mill *et al.*^11^) to 0.99 (95%CI: 0.82–1.19) with significant heterogeneity (P_for heterogeneity_ = 0.194, *I*^2^ = 34.1%) (excluding the study by Turner *et al.*^16^). The Begg rank correlation test (P = 0.133) and Egger linear regression test (P = 0.489) also indicated no evidence of publication bias. Because number of included studies less than 10, we did not perform the funnel plot.

## Discussion

Allergic diseases (immune mediated conditions), encompassing hay fever, allergic asthma and atopy, are caused by inappropriate immunological response to antigens that do not elicit response in most individuals. Allergy-related carcinogenesis is a topic of interest but has generated considerable controversy. There has been a long-standing interest in determining whether individuals with allergic diseases have an altered risk of developing cancer. Several studies investigated the association between allergic diseases and specific cancers. Asthma, hay fever, and atopy have been associated with the risk of several specific cancers, such as pancreatic cancer, lymphomas, brain tumors, breast cancer and leukemia, although inconsistently^10^,^14^,^15^,^46^,^47^. Allergic diseases could theoretically both prevent and induce the development of several specific cancers. Two hypotheses that attempt to explain the possible mechanism between allergic diseases and cancer are immune surveillance and the antigenic stimulation theory^48^. Allergy might enhance the human immune system to recognize and eliminate cancer cells. In contrast, the antigenic stimulation hypothesis proposes that hyperactive immune conditions trigger chronic cellular inflammation, resulting in DNA mutation in dividing cells and inevitably leading to cancer development^49^. The determination of whether asthma, hay fever, atopy and any allergy are associated with prostate cancer has been evaluated in a small number of studies. The small number of prior studies evaluating allergic disease and prostate cancer has not produced consistent results, inverse^12^,^16^,^18^, null^13^,^14^,^15^,^17^,^28^,^29^,^30^,^31^, and positive associations^10^,^11^,^19^,^21^,^22^,^23^,^24^,^25^,^26^,^27^ have been reported. The inconsistent results of previous studies may be due to insufficient study sample size, publication bias, selection bias, lack of adjustment for confounding factors, and the use of different definitions of allergic diseases. A recent meta-analysis of the few studies suggested that asthma (N = 8, pooled RR: 0.93; 95%CI: 0.76–1.15), hay fever (N = 5, pooled RR: 0.96; 95%CI: 0.87–1.05), and any allergy (N = 4, pooled RR: 1.01; 95%CI: 0.87–1.17), but not for atopy (N = 3, pooled RR: 1.43; 95%CI: 1.08–1.91)^20^. However, studies published subsequent to this report have reported inconsistent results^23^,^24^,^25^,^26^,^27^,^28^,^29^,^30^,^31^. In addition, the relation between allergic diseases and cancer risk remain unclear, and appear to be site-specific, we conducted an up-dated meta-analysis to summarize the current proof to evaluate the association between allergic conditions and prostate cancer risk. The meta-analysis suggested that there is little observational support for the two theories in the development of prostate cancer. The effect of hyperreactive state and/or immune surveillance theories maybe mutually offset in the body.

The present meta-analysis exhibited several strengths, compared to the previous published meta-analysis. The first research highlight of this meta-analysis is its large sample size. The large number of total cases provided high statistical power to quantitatively evaluate the association between allergic conditions and prostate cancer risk. In addition, we expand the meta-analysis and the association between different geographic regions and study design, adjusted for covariates was also explored. Second, publication bias is a potential concern in any meta-analysis because small studies with null results do not get published. However, in our meta-analysis, we found little evidence of publication bias.

Nevertheless, there are some several limitations in the present meta-analysis. First, case-control studies have intrinsic limitations, such as selective bias and recall or memory bias. This limitation can partly explained the different results between case-control and cohort studies in the stratified analysis. Second, we cannot exclude the possibility that the observed null relationship between allergic disorders and prostate cancer risk is attributed to confounding factors. Majority of the studies were adjusted for potential confounding factors, but not all potential confounders were adjusted in every study. Although the allergic conditions and prostate cancer share common some potential confounders, such as cigarette smoking, alcohol drinking, old age, BMI and race, in analysis stratified by adjusting the smoking status, age, alcohol drinking BMI and race, similar results were obtained. Third, measurement error in assessment of allergic conditions are known to bias effect estimates, however, none of included studies in the meta-analysis made any corrections for measurement errors. An accurate assessment of allergic conditions remains a challenge, because these measures are based on different assessment methods, such as allergen-specific IgE measurement, skin prick testing, self-reported questionnaire, interviewed by medical staff, and hospital discharge register. Diagnosis of allergic conditions was largely based on a self-reported. Only a few studied were based on measurements that may be condisered to be more objective: serum LgE or skin prick tests. The increasing errors in measurements become inevitable. The imprecise measurement of allergic disorders might have attenuated the true associations. Fourth, potential sources of between-study heterogeneity, which is common in meta-analysis, should be explored although the low heterogeneity was found between hay fever or atopy and prostate cancer risk. Results from subgroup and sensitivity analysis indicated that geographic region, study design, quality of NOS may be potential sources of heterogeneity. Thirds, in subgroup analysis, there was a marginally positive association between hay fever and prostate cancer risk in studies that adjusted for age and alcohol drinking and conducted in the Europe, but a null association among studies adjusted for cigarette smoking and race. Due to the numerous comparisons and few included studies, this positive finding may have been a chance finding. It indicated that more relevant articles are needed to further explore this association. Fifth, analysis of the length of the induction period allows for characterising an exposure–outcome relationship and for falsifying the pathway assumed. The longer the supposed time sequence between the exposure and the occurrence of the outcome, the more crucial to analyze the empirical induction period. The average follow-up ranged from 5.05 years to 43 years among included cohort studies. Patients were followed up over five years in majority of the studies (92.8%). Therefore, the observation period in the included cohort studies covered a reasonable induction period. Overall, these limitations may affect our final conclusions.

In the meta-analysis, we found no indication of an association between allergic conditions (asthma, atopy, hay fever, or any allergy) and risk of prostate cancer, and there is little observational support for the immune surveillace theory or antigenic stimulation theory in the development of prostate cancer risk. However, these results should be carefully interpreted because of the significant heterogeneity among studies and potential confounders. Additional large-scale and high-quality prospective studies are needed to confirm the association between allergic conditions and risk of prostate cancer.

## Methods

### Literature search

A literature search was performed in March 15, 2016 without restriction to regions, publication types, or languages. The primary sources were the electronic databases of Pubmed and Embase databases. To identify eligible studies, the main search employed various combinations of Medical Subject Headings (MeSH) and non-MeSH terms “prostate carcinoma” OR “prostatic cancer” OR “prostate cancer” OR “prostatic carcinoma” combined with “asthma” OR “asthma*” OR “allergy” OR “allerg*” OR “atopy”. The main search was completed independently by two investigators. Any discrepancy was solved by consultation of an investigator, not involved in the initial procedure. Moreover, the reference lists of all the studies and published systematic reviews, meta-analysis were also screened to identify other potentially eligible studies.

### Study selection

To minimize the differences between studies, we imposed the following methodological restrictions for the inclusion criteria: 1) study design of interest was either cohort or case control study 2) the exposure of interest was allergic conditions (asthma, atopy, hay fever and “any allergy”); 3) the outcome was prostate cancer; 4) the study reported enough information to extract effect estimates and the corresponding 95% confidence intervals. In case of multiple publications, only the most recent or comprehensive one was considered eligible. Two authors (JKS and XHY) independently evaluated the eligibility of all retrieved studies and disagreements were resolved through discussion or consultation with a third author (LHG).

### Data extraction

Data from the included studies were extracted and summarized independently by two of the authors using a pre-standardized data extraction form. Any disagreement was resolved by the senior author (ZWD). The following data were extracted from each study: first author, publication year, study design, country, sex, total number of cases and subjects for cohort studies, total number of cases and controls for case-control studies, assessment methods for allergic diseases, quantitative effect estimates (expressed as an odds ratio, hazard risk, relative risk, standardized mortality ratio, or standardized incidence ration) and 95%CI and variables adjusted for in the meta-analysis. The OR in one study^18^ was not extracted, thus, we computed the crude risk estimates and corresponding CI. Two review authors (SJK and ZJG) independently extracted data from the selected studies. Any disagreements were resolved through discussion and consensus.

### Quality assessment

Two review authors (SJK and ZJG) independently assessed the risk of bias using the Newcastle-Ottawa Scale (NOS), which consists of three factors: patient selection, comparability of the study groups, and assessment of outcome. A score of 0–9 (allocated as stars) was allocated to each study^50^,^51^. The studies achieving six or more stars were considered to be of high quality.

### Statistical analysis

Because the incidence of prostate cancer risk was low, the OR/HR was considered as approximation of RR, SMR was also considered as equivalent of SIR^52^. We computed the combined RR and SIR and corresponding 95%CI from the estimated reported in each study. The aggregated results and 95%CIs for effect sizes were calculated using inverse-variance weighted random-effects meta-analysis, which incorporated both within-study and between-study variability^53^. *I*^2^ was used to assess heterogeneity across studies, with *I*^2^ values of 0%, 25%, 50% and 75% representing no, low, moderate and high heterogeneity, respectively. Subgroup analysis was stratified by geographic region, study design, quality of NOS scale, body mass index (BMI) and whether the study adjusted for risk factors, including age, race, alcohol drinking and smoking. The sensitivity analysis was also conducted by removing one study at a time and the rest analyzed to determine whether an individual study affected the aggregate result or not. Small study bias, consistent with publication bias, was evaluated by statistical tests (Begg rank correlation test^54^ and Egger’s linear regression test^55^), the visual examination of funnel plot when the number of included studies ≥10, and the results were considered to indicate publication bias when P < 0.10. All statistical analyses were conducted using Stata version 13.1 (Stata Corp., College Station, TX, USA).

## Additional Information

**How to cite this article**: Zhu, J. *et al.* Association between allergic conditions and risk of prostate cancer: A Prisma-Compliant Systematic Review and Meta-Analysis. *Sci. Rep.* **6**, 35682; doi: 10.1038/srep35682 (2016).

## Figures and Tables

**Table 1:** Characteristic of cohort/nested case-control studies included in the meta-analysis.

Study	Year	Country	Study design	No. of cases	Cohort size	Study period	Age, Median (Range), yrs	Exposure	Exposure assessment	Cancer identification	Follow-up period (years)	Adjustment for covariates
Mcwhorter	1988	USA	A prospective cohort study (NHANESI)	34	6108	1971–1984	NA(25–74)	Any allergy	The physician-diagnosed allergy	Hospital or nursing home records or on death certificate	10	Adjusted for age, race, and smoking status.
Mills	1992	USA	A cohort study	180	34,198	1977–1982	NA	Asthma; hay fever; any allergy	A detailed life-style questionnaire on medical history	Medical records and cancer registries	6	Adjusted age, smoking history, and time since last physician contact.
Kallen	1993	Sweden	A cohort study	671	64,346	1969–1983	NA	Asthma	Hospital discharge register	Swedish cancer registries and death registries	14	NA
Vesterinen	1993	Finland	A cohort study	256	77,952	1970–1987	NA(35–84)	Asthma	Finish social insurance institution’s file of asthma patients	Finnish cancer registry	17	NA
Eriksson	1995	Sweden	A cohort study	1	6,593	1976–1989	31(16–80)	Asthma	Skin prick tests;	Swedish national tumor registries	13	NA
Talbot-Smith	2002	Australia	A prospective cohort study	86	1,522	1981–1999	Men:50.7;Women:50.0	Asthma; hay fever; atopy	Self-reported physician-diagnosed allergy and skin prick testing	Linkage to death registrations and Australian cancer registry	8	Adjusted for age, smoking status, and body mass index.
Gonzalez-Perez	2005	Spain	A nested case-control study	407	9,488	1994–2000	NA(20–79)	Asthma	Automated general practitioner records	Automated general practitioner records	6	Adjusted for age, calendar year, BMI, alcohol intake, smoking status, prior comorbidities (cardiovascular disease, diabetes, osteoarthirtis, and rheumatoid arthritis), health services utilization, use of aspirin, NSAID, and paracetamol using logistic regression.
Turner	2005	Canada	A prospective cohort study	5,674 (mortality)	1,102,247	1982–2000	NA(≥30)	Asthma; hay fever; any allergy	Self-reported physician-diagnosed allergy	National death index	18	Adjusted for race, smoking, education, body mass index, exercise, alcohol drinking, family history of prostate cancer, and consumption of vegetables, fat, and red meat.
Ji	2009	Sweden	A cohort study	1008	140,425	1965–2004	NA	Asthma	The Swedish hospital discharge register	Cancer register-identified cases	40	NA
Severi	2010	Australia	A prospective cohort study (Melbourne Collaborative Cohort Study (MCCS))	1,179	16,394	1990–2007	NA(27–81)	Asthma	A structure interview schedule and history of medical conditions	Australia state cancer registry	13.4	Adjusted for age, country of birth, education, body mass index, fat and fat-free mass, smoking, alcohol consumption, and total energy intake.
Hemminki	2014	Swede	A cohort study	404	138,723	1964–2010	NA	Hay fever/allergic rhinitis	Hospital Discharge Register	Swedish Cancer Registry	NA	NA
Skaaby	2014	Danish	A prospective population-based cohort study	175	14,849	1976–2008	Nonatopic:46.3 ± 10.9; Atopic 43.7 ± 10.4	Atopy	Serum specific IgE positivity measurements	Danish cancer registry	11.8	Adjusted for age (age is underlying time axis), education, physical activity, smoking habits, alcohol intake, and BMI.
Platz	2015	Sweden	A prospective population-based cohort study (Health Professionals Follow-up Study)	6294	47, 880	1986–2012	NA(40–75)	Asthma; hayfever	Queationnaire	Medical and pathology reports (90%)	26	Adjusted for age, race (African-American, Asian, other ancestry and white), first-degree family history of prostate cancer, height (inches), body mass index (BMI, kg/m2, updated), BMI at the age of 21 years (kg/m2), vigorous physical activity (MET-hours/week, updated), diabetes (updated) and cigarette smoking in the past 10 years (pack-years, updated).
Su	2015	Taiwan (China)	A population-based case-cohort study	74	12,372	1997–2008	54.87(15–65^+^)	Asthma	Medical claims data	Prostate cancer claim records and histopathologic findings or significantly elevated prostate-specific antigen with radiologic evidence of metastasis	5.05	Adjusted for age, residential area, insurance premium, hypertriglyceridemia, hypertension, diabetes mellitus, chronic obstructive pulmonary disease, duration of hospitalization, and mortality.
Taqhizadeh	2015	Netherlands	A general population-based cohort study	83(mortality and hospitalization)	8,465	1965–2008	NA(20–65)	Asthma	Peripheral blood eosinophil counts; Skin prick tests; Serum total Immunoglobulin E (IgE)	Hospital admission register	43	Adjusted for age, Forced Expiratory Volume in 1 s (FEV 1) as % of predicted, BMI (all at the first survey), and place of residence.

**Table 2:** Characteristic of case-control studies included in the meta-analysis.

Study	Year	Country	Study design	No. of cases	No. of controls	Study period	Age, Median (Range), yrs	Exposures	Exposure assessment	Cancer identification	Adjustment for covariates
Vena	1985	USA	A retrospective case-control study	263	1562	1957–1965	NA	Asthma, hay fever	Self-completed questionnaire and medical history interview	Admissions with cancer	Adjusted for age and smoking.
Ohrui	2002	Japan	A case-control study	10	1202	1995–2000	Case:68; control:69	Asthma	Clinical diagnosis	Clinical diagnosis	NA
Wang	2006	Germany	A case-control study	318	1904	2000–2003	NA(50–74)	Asthma, hay fever, atopy, any allergy	Allergen-specific LgE measurement; questionnaire on physician diagnosed allergy	Histologically confirmed cancers	Adjusted for age, education, body mass index, family history of cancer (first degree), smoking status and alcohol consumption.
El-Zein	2010	Canada (Montreal)	A population-based case-control study	397	512	1979–1986	NA(35–70)	Ashtma, eczema	Self-reported history of medical conditions	Histologically confirmed cancers.	Adjusted for age, income, respondent status, ancestry, smoking, and farming.
Weiss	2014	Canada (Montreal)	A population-based case-control study (Montreal PROtEuS study)	1936	1995	2005–2009	Case:63.6; Control 64.8	Asthma, allergy; hay fever	Self-reported asthma and allergy	Que´becn Tumour registery (80%)	Adjusted for age, ancestry and familial history of prostate cancer.

**Table 3:** Quality assessment of eligible studies based on Newcastle-Ottawa scale.

Author	year	Selection	Comparability	Exposure
Vena	1985	2	1	2
Mcwhorter	1988	3	1	2
Mills	1992	3	1	1
Kallen	1993	3	0	2
Vesterinen	1993	3	0	2
Eriksson	1995	3	0	2
Ohrui	2002	3	0	2
Talbot-Smith	2002	3	1	2
Lightfoot	2004	3	1	2
Turner	2005	3	2	2
Gonzalez-Perez	2005	3	2	2
Wang	2006	3	2	2
Ji	2009	3	0	2
Severi	2010	3	2	2
Hemminki	2014	3	0	2
Weiss	2014	3	1	2
Skaaby	2014	3	2	2
Platz	2015	3	2	2
Su	2015	3	2	2
Taqhizadeh	2015	3	2	2

**Table 4:** Results of subgroup analysis of asthma, hay fever, atopy, and any allergy.

Subgroup analysis	Asthma				Hay fever				Atopy				Any allergy			
	**N**	RR(95%CI)	I2(%)	P value	**N**	RR(95%CI)	I2(%)	P value	**N**	RR(95%CI)	I2(%)	P value	**N**	RR(95%CI)	I2(%)	P value
Total	13	1.04(0.92–1.17)	73.2	0.000	6	1.04(0.99–1.09)	0.0	0.428	3	1.25(0.74–2.10)	73.2	0.024	6	0.96(0.86–1.06)	19.3	0.287
Study design																
Cohort study	8	1.02(0.91–1.15)	68.0	0.003	3	1.03(0.93–1.14)	56.1	0.103	2	1.31(0.43–4.01)	81.9	0.019	3	1.06(0.84–1.33)	48.9	0.142
Case-control study	5	1.20(0.69–2.09)	82.5	0.000	3	1.02(0.76–1.36)	0.0	0.848	1	1.35(1.00–1.83)	NA	NA	3	0.88(0.75–1.04)	0.0	0.497
Geographic region																
North America	5	0.95(0.81–1.12)	20.9	0.281	4	0.97(0.88–1.06)	0.0	0.699					5	0.96(0.84–1.10)	35.2	0.187
Europe	4	0.90(0.81–1.00)	0.0	0.698	2	1.07(1.01–1.09)	0.0	0.522	2	1.05(0.62–1.77)	77.2	0.036	1	0.98(0.66–1.45)	NA	NA
Asia	2	4.55(0.23–89.94)	94.3	0.000												
Australia	2	1.36(0.98–1.90)	32.9	0.222					1	2.49(1.04–5.93)	NA	NA				
NOS score																
High	10	1.02(0.93–1.13)	61.4	0.006	4	1.02(0.94–1.11)	33.2	0.213	3	1.25(0.79–2.10)	73.2	0.024	5	0.93(0.86–1.00)	0.0	0.612
Low	3	1.90(0.29–12.61)	90.6	0.000	2	1.15(0.78–1.70)	0.0	0.715					1	1.25(0.93–1.69)	NA	NA
Adjusted for confounders or important risk factors Age																
yes	10	1.02(0.88–1.18)	55.6	0.016	5	1.07(1.01–1.13)	0.0	0.951	2	1.59(0.93–2.71)	41.0	0.193	5	0.99(0.82–1.19)	34.1	0.194
no	3	1.04(0.92–1.17)	73.2	0.000	1	0.95(0.86–1.05)	NA	NA	1	0.79(0.53–1.18)	NA	NA	1	0.94(0.86–1.02)	NA	NA
Race																
yes	2	0.90(0.81–1.00)	0.0	0.769	4	1.07(0.84–1.36)	0.0	0.872					2	0.94(0.87–1.03)	0.0	0.380
no	11	1.10(0.93–1.29)	69.5	0.000	2	1.02(0.90–1.14)	75.9	0.042	3	1.25(0.74–2.10)	73.2	0.024	4	0.97(0.79–1.18)	44.4	0.145
Cigarette smoking																
yes	8	0.99(0.84–1.17)	62.7	0.009	5	1.03(0.96–1.00)	17.5	0.303	3	1.25(0.74–2.10)	73.2	0.024	3	1.06(0.84–1.33)	48.9	0.142
no	5	1.17(0.88–1.56)	79.2	0.001	1	1.09(0.74–1.61)	NA	NA					3	0.88(0.75–1.04)	0.0	0.497
Alcohol drinking																
yes	9	1.07(0.89–1.28)	77.2	0.000	4	1.07(1.01–1.13)	0.0	0.963	2	1.05(0.62–1.77)	77.2	0.036	5	0.99(0.82–1.19)	34.1	0.194
no	4	1.04(0.83–1.24)	65.8	0.032	2	0.95(0.85–1.05)	0.0	0.843	1	2.49(1.04–5.93)	NA	NA	1	0.96(0.86–1.06)	NA	NA
BMI																
yes	7	1.02(0.87–1.20)	62.8	0.013	3	1.01(0.92–1.12)	0.0	0.916	3	1.25(0.79–2.10)	73.2	0.024	2	0.94(0.87–1.02)	0.0	0.839
no	6	1.08(0.80–1.45)	78.2	0.000	3	1.12(0.85–1.48)	54.9	0.109					4	1.00(0.79–1.26)	50.5	0.108
